# A case of de novo psoriasis secondary to atezolizumab in a patient with hepatocellular carcinoma

**DOI:** 10.1002/kjm2.12595

**Published:** 2022-09-30

**Authors:** Jiong‐Huang Lim, Yang Lo

**Affiliations:** ^1^ Department of Dermatology Cathay General Hospital Taipei Taiwan; ^2^ School of Medicine, College of Medicine Fu Jen Catholic University New Taipei Taiwan

The use of immune checkpoint inhibitors (ICIs) is increasing nowadays because of their highly selectivity for to several cancer types. The targets of ICIs are CTLA‐4 (cytotoxic T‐lymphocyte‐associated protein 4), programmed cell death protein 1 (PD‐1), and the ligands of PD‐1 (PD‐L1). Cutaneous adverse reactions are one of the most common immune‐related adverse reactions. Maculopapular rash and pruritus are the most common manifestations of these reactions although but lichenoid reactions, eczema, and vitiligo were also reported as immune‐related adverse events.^1^ Exacerbated and de novo of psoriasis were mostly reported with the use of nivolumab and pembrolizumab^2^ and were rarely associated with the use of atezolizumab. Herein, we present a case involving the new onset of psoriasis vulgaris in a patient treated with atezolizumab.

A 68‐year‐old man with a history of hepatocellular carcinoma presented with multiple itchy erythematous plaques on the lower extremities. The rash developed after the patient underwent treatment with 1200‐mg atezolizumab plus 500‐mg bevacizumab for 1–2 weeks. The plaques mostly resolved in the first week after the application of fluocinonide cream 0.05% and then disappeared spontaneously.

Three weeks after the patient underwent a second treatment course with atezolizumab and bevacizumab, the patient experienced the recurrence of extensive erythematous plaques within 2 weeks. A physical examination revealed multiple, well‐demarcated, erythematous plaques with a whitish scaly surface on the buttocks and extremities (Figure 1A,B). A skin biopsy from the inner thigh indicated psoriasiform dermatitis with lymphocytes and eosinophils infiltration. Mounds of parakeratosis and focal spongiform pustule of Kogoj (Figure 1C,D) were also identified. The results of periodic‐acid‐Schiff staining were negative. On the basis of the clinical and histopathological features of the patient, drug‐related psoriasiform eruption was diagnosed. After a discussion with an oncologist, atezolizumab was determined to be the most likely culprit drug. The drug was discontinued, and the lesions disappeared gradually. Thus, atezolizumab‐induced psoriasis was considered to be the cause of the lesions.

**FIGURE 1:**
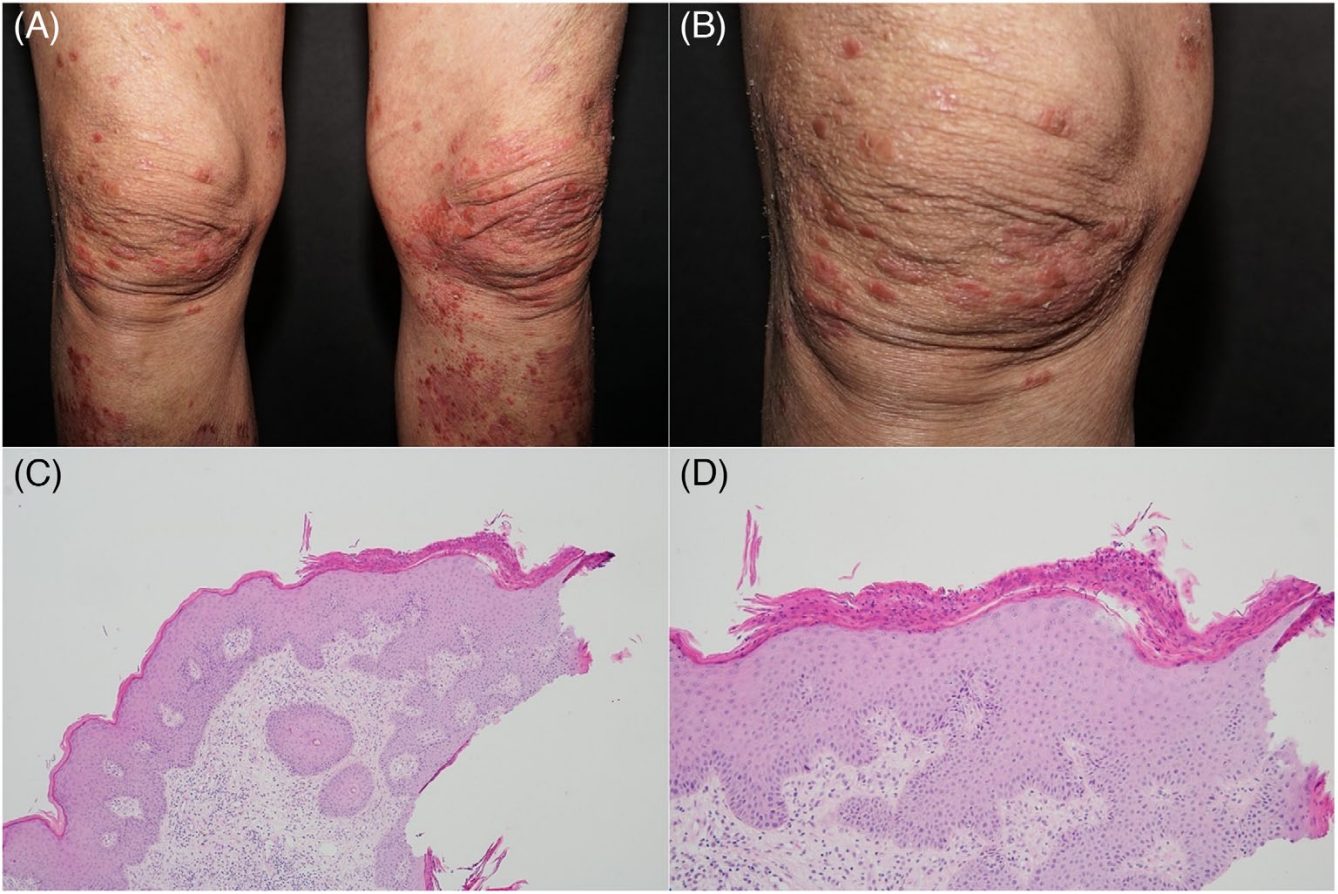
(A) Clinical images of several well‐demarcated erythematous papules on the lower legs. (B) Clinical image: close inspection reveals psoriasiform pattern. (C) Histopathological image: skin biopsy specimen from the thigh showed psoriasiform proliferation and lymphocytic infiltration (hematoxylin–eosin stain: magnification, ×100). Histopathological image: noted mound of parakeratosis and focal spongiform pustules of Kogoj (hematoxylin–eosin stain: magnification, ×200)

ICIs are widely used for treating advanced malignancies. The most common cutaneous adverse events associated with ICIs are maculopapular rashes and pruritus, followed by lichenoid dermatitis, vitiligo, and psoriasis.^1^ Psoriasis was reported to be a side effect in patients who were treated with nivolumab and pembrolizumab. De novo psoriasis resulting from atezolizumab is rare (Table 1).^3^ Notably, all reported cases have involved male patients, including our case. The pathogenesis of PD‐1/PD‐L1 in the development of psoriasis is still unclear. Researchers have demonstrated that anti‐PD‐1 can inhibit Treg activity, which influences the pathogenesis of psoriasis.^4^ The temporal relationship between the initiation of atezolizumab treatment and the onset of psoriasis, the improvement observed after discontinuation, and the recurrence after a rechallenge all suggest a correlation between psoriasis and atezolizumab. Because of the limited number of reported cases, estimates of the time between medication administration and psoriasis occurrence vary, the average estimate is 12 weeks after ICI initiation.^4^ Psoriasis can be managed using topical glucocorticoids or vitamin D3 analogs. Phototherapy, retinoids, methotrexate, and apremilast are verified options that may be prescribed according to the severity of psoriasis.^5^ The discontinuation of immunotherapy is not required for most patients.^5^ However, the literature indicates that lesions usually heal rapidly after the discontinuation of immunotherapy.

**TABLE 1:** Reported cases of atezolizumab‐induced psoriasis

Author	Sex	Age	Indication for atezolizumab	Onset after atezolizumab usage	Duration of disease	Response to therapy
Paola Corneli et al. (2020)	M	76	Metastatic NSCLC	1 week	Not mention	Lesions healed after discontinued atezolizumab
Yuri Onishi et al. (2021)	M	75	NSCLC	10 months	2 months	Lesion healed after discontinued atezolizumab
Alpana et al. (2022)	M	40	Advanced urothelial carcinoma	2 months	Not mention	Lesion healed under oral acitretin and topical salicylic acid and clobetasol
Shigeruko et al. (2019)	M	70	Advanced squamous NSCLC	After third round of treatment (3 weeks as one course)	24 weeks	Lesion healed under calcipotriol/betamethasone dipropionate ointment

Abbreviations: M, male; NSCLC, non–small cell lung cancer.

In conclusion, we report a rare case of de novo psoriasis vulgaris that developed as a side effect of atezolizumab treatment in a patient with hepatocellular cancer. Early recognition and adequate management of side effects are crucial because they enable ICI therapy to continue without interruption.

## CONFLICT OF INTEREST

The authors declare no conflict of interest.
